# Chronic itch induced by thalamic deep brain stimulation: a case for a central itch centre

**DOI:** 10.1186/s12967-021-03110-y

**Published:** 2021-10-16

**Authors:** Luciano Furlanetti, Harutomo Hasegawa, Natasha Hulse, Rachael Morris-Jones, Keyoumars Ashkan

**Affiliations:** 1 Department of Neurosurgery, King’s College Hospital NHS Foundation Trust, Denmark Hill, London, SE5 9RS UK; 2 Department of Dermatology, King’s College Hospital NHS Foundation Trust, London, UK; 3 King’s Health Partners Academic Health Sciences Centre, London, UK

**Keywords:** Neuropathic pain, Deep brain stimulation, Thalamus, Central itch

## Abstract

**Background:**

Central itch syndrome has been previously described in conditions such as stroke. The neurophysiology of central itch syndrome has been investigated in non-human primates but remains incompletely understood.

**Methods:**

We report an observational study of a rare case of severe central itch following thalamic deep brain stimulation and postulate the location of the central itch centre in humans.

**Results:**

The patient was a 47-year-old female, with congenital spinal malformations, multiple previous corrective spinal surgeries and a 30-year history of refractory neuropathic pain in her back and inferior limbs. Following multidisciplinary pain assessment and recommendation, she was referred for spinal cord stimulation, but the procedure failed technically due to scarring related to her multiple previous spinal surgeries. She was therefore referred to our centre and underwent bilateral deep brain stimulation (DBS) of the ventral posterolateral nucleus of the thalamus for management of her chronic pain. Four weeks after switching on the stimulation, the patient reported significant improvement in her pain but developed a full body progressive itch which was then complicated with a rash. Common causes of skin eczema were ruled out by multiple formal dermatological evaluation. A trial of unilateral “off stimulation” was performed showing improvement of the itchy rash. Standard and normalized brain atlases were used to localize the active stimulating contact within the thalamus at a location we postulate as the central itch centre.

**Conclusions:**

Precise stereotactic imaging points to the lateral portion of the ventral posterolateral and posteroinferior nuclei of the thalamus as critical in the neurophysiology of itch in humans.

## Introduction

Itch is an irritative sensation, usually originating in the skin, but also in transitional tissues, such as conjunctiva or mucosa [1]. It is understood as a protection against harmful agents, leading to an unpreventable desire to scratch the region affected [1–3]. A network of sensory receptors and nerves are able to discriminate not only between touch, pressure, heat and cold, but also nociceptive stimuli causing pain and itch [1, 4].

Itch can be generally classified as systemic, infectious, dermatological, medication-related, psychiatric or neuropathic [2]. Interestingly, although the classic mediator of itch is histamine, in many conditions other than classic urticaria, it cannot be effectively managed with antihistamines, indicating the involvement of nonhistaminergic pathways, mediated through activity in both unmyelinated and myelinated afferents [5, 6]. Scientific evidence derived from clinical and preclinical studies support the concept that pain and itch neural networks interact and possibly overlap to some extent, suggested for instance, by similar stinging and burning paresthesia reported in poststroke central neuropathic itch and also in neuropathic pain [3, 7]. Furthermore, it is well-known that a spinal cord injury or a therapeutic lesion of the lateral spinothalamic tract (STT)—a.k.a. cordotomy—may lead to loss of temperature, pain, and itch sensations, due to the interruption of shared neural pathways. On the other hand, the same way that pain (for instance, caused by scratching) relieves itch, the use of opiates for pain treatment may cause itch, denoting an intrinsic functional relationship between these complex sensory networks.

Acute itch is experienced when a pruritogenic stimulus contacts the surface of the skin and is relieved by scratching, which activates spinal interneurons to inhibit itch-transmitting neurons [1, 3, 8]. Chronic itch, on the other hand, is defined as a persistent sensation of pruritus, which does not improve with scratching [1], and involves sensitization mechanisms of itch-signaling and dysfunction of itch-inhibitory pathways [2–4, 9]. Neuropathic itch (NI) is a form of chronic pruritus that occurs in the absence of pruritogenic stimuli [10]. NI may develop following damage to itch-neural networks at any level. Possible reported causes of NI are, for instance, stroke, infectious diseases, traumatic injury, tumors, demyelinating disorders, myelopathy, radiculopathy and polyneuropathy [8, 11–18]. Therefore, chronic NI is the consequence of a network disorder caused by imbalances between excitatory and inhibitory neural signaling rather than lesion in one specific location, although specific nodes within the network may play crucial roles [9, 10]. Central itch has been more often associated with lesions involving the lateral medulla (Wallenberg’s syndrome), rostral lateral pons and posterior thalamic/retrolenticular areas (Dejerine-Roussy syndrome) [8, 10]. Since, in most such cases intrusive neuropathic pain becomes the main focus of attention, itch of central origin might be overseen or even mistakenly interpreted as mild central pain, and therefore, rarely specifically investigated.

Here, we report a rare case of severe central itch following thalamic deep brain stimulation for the management of neuropathic pain. By means of detailed stereotactic imaging available and review of state-of-art works on the neurophysiological mechanisms underpinning pain and itch, we postulate the location of the thalamic central itch centre in humans.

## Methods

### Background

The patient was a 47-year-old female, with congenital spinal malformations and multiple previous corrective spinal surgeries during adolescence. She had a 30-year history of complex regional pain syndrome with refractory neuropathic pain in her both legs, particularly in the left lower limb, and failed-back surgery syndrome following spinal stabilization procedures. The patient would describe two types of pain: (i) a moderate to severe (VAS 7–9), continuous, stabbing and burning pain in the lumbosacral region, which increased by standing and walking, and did not settle completely when resting; (ii) permanent numbness associated with a shooting pain in the lower limbs, mainly on the left side and down to her feet. Clinical and neuroimaging investigation ruled out any neuronal compression, infection, or any signs of instability of the lumbar spine. She was on high dose analgesic medications, including opiates. Apart from chronic pain, she had type 2 diabetes mellitus and asthma. Despite her moderate disability due to refractory back and leg pain, she was mobile with walking-aids and able to perform routine domestic tasks with help. Following multidisciplinary pain assessment and recommendation, she was referred for spinal cord stimulation, but the procedure failed technically due to scarring related to her multiple previous spinal surgeries. She was therefore referred to our centre and underwent bilateral deep brain stimulation (DBS) of the ventral posterolateral nucleus of the thalamus for management of her chronic neuropathic back and leg pain.

### Surgical procedure

Bilateral octopolar DBS electrodes (Vercise, Boston Scientific Corp., USA) were implanted under local anaesthesia using stereotactic MRI and the Leksell G frame (Elekta, Stockholm, Sweden). The electrodes were connected to a single rechargeable implantable pulse generator (Vercise Gevia, Boston Scientific Corp., USA) placed subcutaneously in the infraclavicular region in a single-stage operation. The final position of the electrodes was verified by fusion of the postoperative stereotactic CT with the pre-operative stereotactic planning MRI scan. Targeting for DBS electrode placement was planned based on the somatotopic organization of the homunculus within the VPL, from medial—head representation, to lateral—lower-extremity, with the upper extremity between based on the Schaltenbrand and Wahren Atlas [19]. Since patient’s neuropathic pain was concentrated in the lower back and legs, the lateral portion of the posterolateral thalamic complex was aimed. The anatomical starting point for the target was then refined by intra-operative test stimulation, aiming for paresthesia to cover the painful body parts of interest. The stereotactic coordinates of the final position of the active stimulating contacts in relation to the mid-commissural point (MCP), were: (right) lateral 16.33 mm, antero-posterior-8.50 mm and vertical-2.96 mm; (left): lateral-17.40 mm, antero-posterior-7.40 mm and vertical-1.18 mm, respectively*.*

### Clinical evaluation

**Fig. 1:**
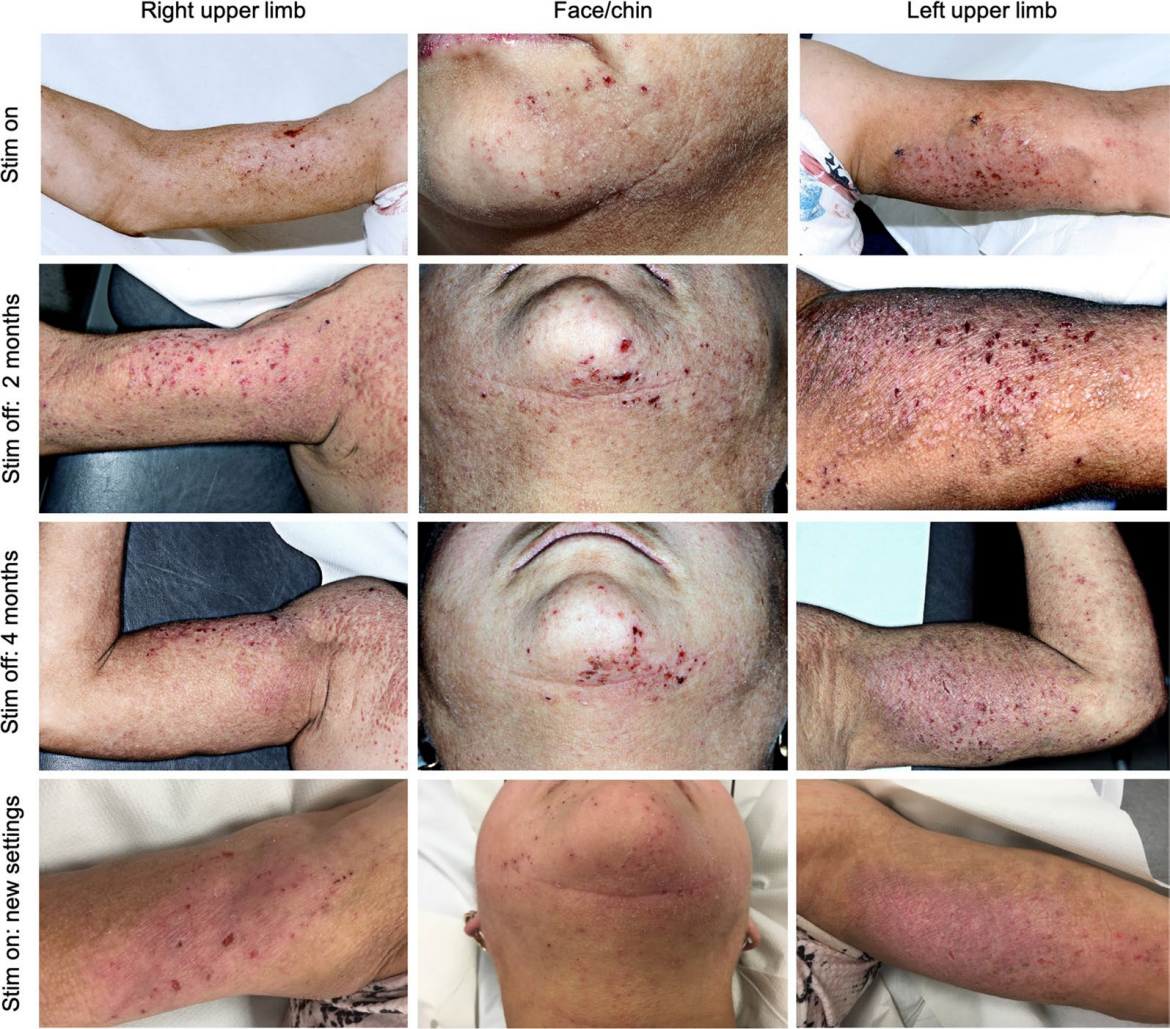
Clinical presentation and evolution of DBS-induced central itch. Upper row (Mai/2018): pronounced active skin inflammation, lichenification with arcuate and serpiginous areas on upper limbs and face caused by intense pruritus after DBS was switched on. Upper middle: reduced inflammation on the right-hand side of the face and body 2 months after the left DBS lead had been switched-off. Of note, improvement of itch and scratch lesions on the right-hand side occurred in detriment of pain control. Botton middle row: itch symptoms on the right-hand side stabilized following 4 months with the left DBS off switched-off. Since pain was uncontrollable, DBS was switched on bilaterally, however using different stimulation settings. Bottow row: 12-month follow-up after fine-tuning of DBS settings, presenting remarkable improvement of pruritus and skin inflammation on both sides

Four weeks after switching the stimulation on, the patient reported beneficial effect on her pain control, with at least 50% overall improvement on VAS scale and also of her mobility due to better pain control. Nevertheless, concomitant with pain relief, the patient reported significant itch on her arms and legs, which had appeared quite suddenly, following stimulation (Fig. 1). The initial stimulation parameters were as follows: [(left electrode: Channel 1, Case + , 1-, 30 Hz, 80 ms, 2.0 mA), (right electrode: Channel 2, Case + , 9-, 10 Hz, 80 ms, 2.0 mA)]. Common causes of skin eczema were ruled out by formal dermatological evaluation, including skin biopsies, that showed a form of nonspecific psoriasiform spongiosis. She continued to be managed by her local dermatologists with a range of medications including methotrexate, azathioprine, and cyclosporine over a 4-year period but with unsatisfactory response. Thus, she was referred back to our multidisciplinary team and following sub-specialist dermatology advice at our centre, the patient was consented for and agreed to be submitted to a 4-month trial of stimulation off, as an attempt to evaluate the hypothesis that the deep brain stimulation drove her itch centrally. Given the severity of her pain, we agreed to turn off the stimulation unilaterally (left brain electrode, corresponding to the right hemibody) which allowed the patient to continue to have some pain relief. This also enabled us to utilize the left hemibody, corresponding to the “on” electrode, as the control.

The patient was regularly assessed and objectively evaluated by neurologists, neurosurgeons and dermatologists during the trial, and the evolution of the skin lesions documented by medical photography. The Pruritus Severity Scale (PSS) and the Dermatology Life Quality Index (DLQI) were applied in every follow-up consultations [20, 21]. The PSS consists of a 12-question validated assessment tool, where several clinical features of pruritus in patients’ daily life, such as intensity, extent, duration, influence on concentration and psyche are evaluated. The 10-question DLQI concerns patient’ perception of the impact of skin diseases on different aspects of their health-related quality of life over a period of time [21].

### Neuroradiological assessment

Stereotactic planning MRI sequences were obtained using a 1.5 T General Electric (GE) MRI scanner (GE Healthcare, Chicago, Il, USA) preoperatively. MRI sequences consisted of volumetric T1-weighted and 2 mm thick contiguous through target T2-weighted axial slices [22]. Postoperative stereotactic CT was linearly co-registered to pre-operative planning MRI sequences using FLIRT (Linear Image Registration Tool) implemented in FSL (Functional MRI of the Brain’s Software Library, University of Oxford) [23]. Pre- (and post-) operative acquisitions were spatially normalised into MNI_ICBM_2009b_NLIN_ASYM space based on preoperative acquisitions, using the SyN registration approach with Advanced Normalization Tools [24]. Nonlinear deformation into template space was achieved in five stages, as previously detailed elsewhere [24]. Co-registrations were manually controlled and refined if needed. DBS electrodes were first automatically pre-localised in native and template space using the PaCER algorithm and then manually localised based on post-operative acquisitions using a tool specifically designed for this task in the Lead-DBS software [25]. Lead-DBS is a MATLAB-based software (MathWorks Inc, USA) package whose code is available at GitHub (www.github.com/netstim/leaddbs) [24]. Active electrode contacts were then localised, and volume of tissue activated (VTA) modelled based on patient-specific stimulation parameters. VTA estimation followed the concepts previously described elsewhere [24]. Using VTAs as seed regions, structural connectivity estimates were computed using a structural connectome [24], which consisted of high-density normative fibre tracts based on 32 healthy subjects of the Human Connectome Project at Massachusetts General Hospital (http://ida.Ioni.usc.edu/login.jsp). Automated segmentation of the Thalamus was employed as defined by the DISTAL atlas [26], whereas brain cortical parcellation was applied based on the Hammersmith brain atlas [27]. Parcellations were manually refined when any mismatches following co-registration were found.

**Fig. 2:**
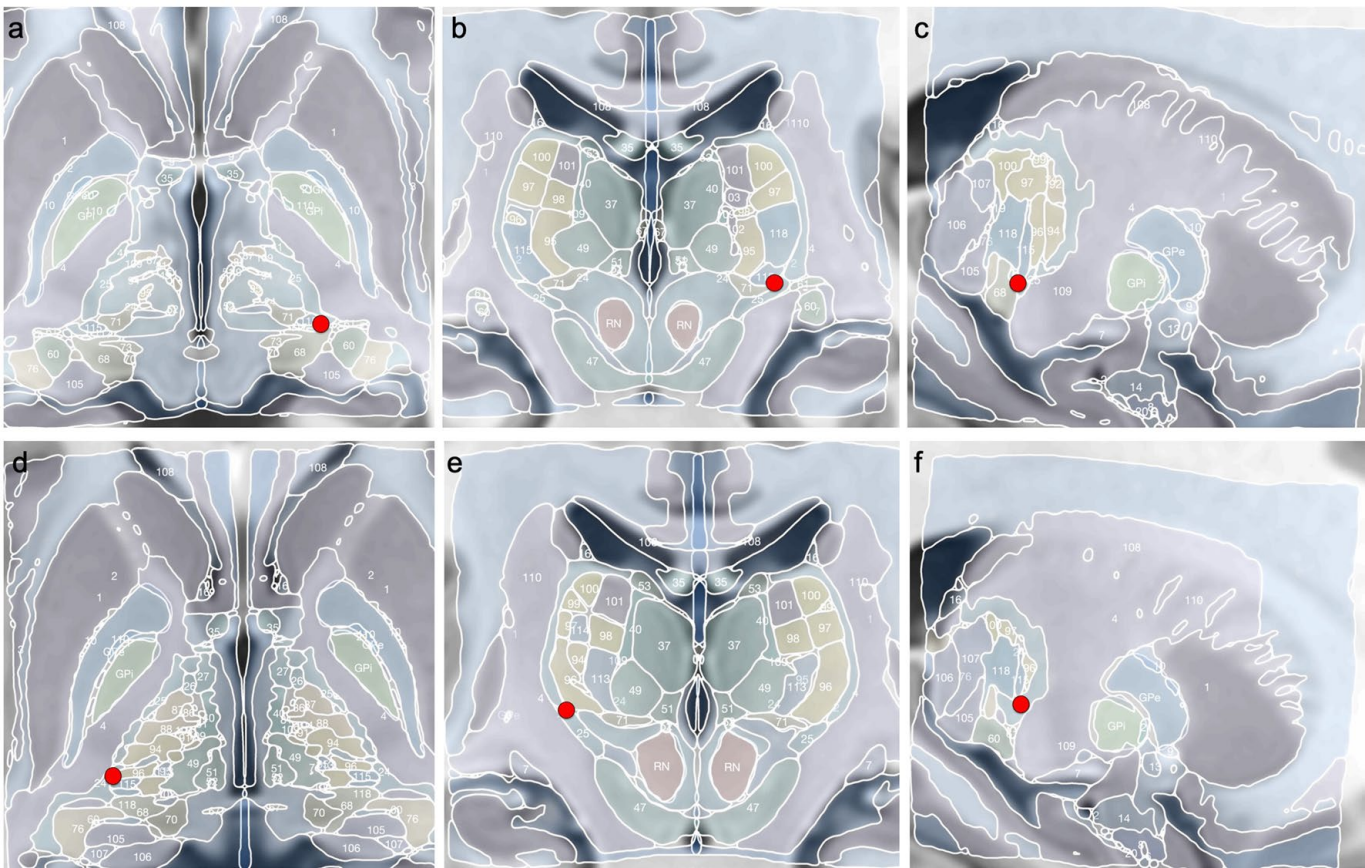
Co-registration and normalization of patient’s scans to MNI space. Upper row (**a**: axial, **b**: coronal, **c**: sagittal) Localization of the left active contact (red dots) in the borders of the following thalamic nuclei: lateral part of the ventral posterolateral nucleus, ventral posteroinferior nucleus and reticular nucleus. Lower row (**d**: axial, **e**: coronal, **f**: sagittal) right active contact (red dots), bordering the lateral part of the ventral posterolateral nucleus, ventral posteroinferior nucleus and the internal capsule

An ultra-high resolution single-echo multi-flip Fast Low-Angle Shot (FLASH) MRI dataset spatially normalised into MNI_ICBM_2009b_NLIN_ASYM space was used as template in Fig. 2 [28]. Standard and normalized brain atlases were used to localize the active stimulating contacts within the thalamus at a location we postulate as the central itch centre (Fig. 2).

## Results

The patient underwent a trial of unilateral “stimulation off” for 4 months, with worsening of her original neuropathic back and leg pain, but gradual improvement of the itchy rash in the corresponding hemibody. The dermatological objective assessment of her pruritus revealed 36.8% improvement on PSS (from initial 19 to 12 points) and 72% on the DLQI (from initial 25 to 7 points). Due to the severity of her pain, bilateral stimulation was finally switched on with optimization of the stimulation settings. Following adjustments of the stimulation parameters, itch symptoms were minimal, whilst neuropathic pain was kept under satisfactory control [stimulation settings on last review a year later: (left electrode: Channel 1, Case + , 1-, 40 Hz, 80 ms, 1.0 mA), (right electrode: Channel 2, Case + , 9- 10-, 40 Hz, 80 ms, 1.0 mA)].

**Fig. 3:**
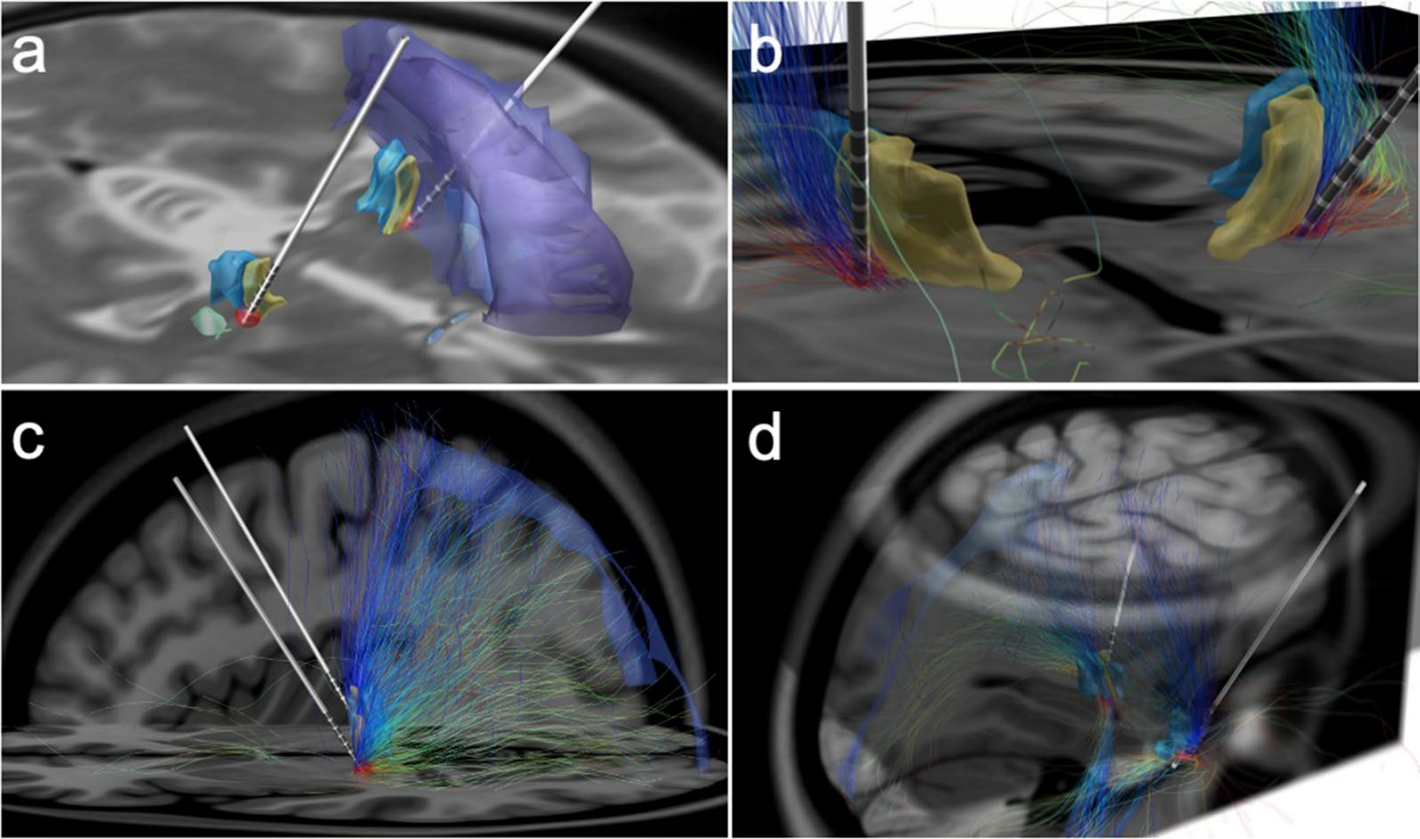
Perspective view of the estimated volume of tissue activated (VTA) in relation to the involved thalamic nuclei (**a**). Tractography (**b**) and connectivity study of the left hemisphere (**c**, **d**) revealing fibers passing through the VTA with cortical projection to the primary sensory cortex and the posterior cerebral quadrant (light blue area overlaid on normalized T1 MRI scan). VTA (red spheres), ventral posterolateral nucleus anterior (yellow); ventral posterolateral posterior (blue); medial lemniscus (light yellow)

**Fig. 4:**
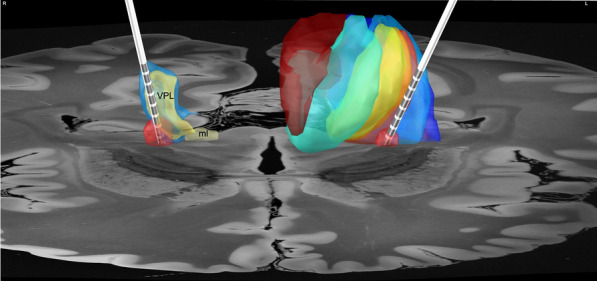
3D Reconstruction of the DBS electrodes co-registered into MNI space. Right side (R), depiction of the relationship of the VTA within the ventral posterolateral complex (ventral posteroinferior nucleus underneath). Left side (L) connectivity atlas of the thalamus, showing the active contact within the area (light red and blue) correspondent to thalamic nuclei (ventral posterior and lateral complex) with projections to somatosensory brain regions, according to Behrens et al. [29]

Interestingly, the neuroradiological analysis of the position of the electrodes in the sensory thalamus, revealed the active contacts to sit within the targeted ventroposterior/ventrobasal thalamic complex. On the left side, it was bordering the posterior part of the ventral posterior lateral (VPLp), ventral posterior inferior (VPI), ventral posteromedial (VPM) and the reticular nucleus of the thalamus. On the right side, the active contact was on the lateral border of the ventral posterior lateral nucleus, close to the posterior limb of the internal capsule (Fig. 3a–d). Furthermore, based on the initial stimulation settings, the estimated volume of tissue activated (VTA) did also partially encroach adjacent structures, such as the zona incerta (bottom), the lemniscus medialis (medial), and the posterior limb of the internal capsule (lateral) (Fig. 3a–d). Connectivity analysis revealed strong projection of the streamlines passing through the VTA, in the midbrain, to the primary and secondary somatosensory cortices (Fig. 4) [29].

Due to limited pain control during the stimulation-off trial, decision was taken to switch the DBS device back on. Optimization of the stimulation settings, avoiding spread of current beyond the ventral posterior thalamic complex, allowed considerable relief of pain, without recurrence of pruritus.

## Discussion

Central itch may pose a challenge both for attending dermatologists and neurologists. This is partly due to the rarity of the condition and partly as a result of late presentation to specialist services, with many patients having already received a range of empirical therapies which might mask or complicate the initial signs and symptoms. In our patient, for several years, the presentation was inaccurately interpreted by the local team as a primary skin rash which had turned itchy, rather than the primary sensation of an irritating itch driving scratching and the subsequent rash.

Although not very often seen as a manifestation of neurological conditions, pruritus of central origin has been previously described in association with both central and/or peripheral nervous system injuries of diverse etiologies, such as infection, tumors, demyelinating disorders, and more frequently, stroke [8, 11–13, 15–17]. Nevertheless, we report a rare presentation of central itch induced by thalamic deep brain stimulation for the management of refractory neuropathic pain.

Acute pain and itch represent two distinct sensations, with different underlying neural networks and behavioral responses (scratching versus withdraw). Although, evidences from preclinical works support the existence of neural discrimination between itch and pain, the neurobiology of chronic itch and pain share largely similar mechanisms [1, 8–10, 13], common pathways and closely related thalamic relays [3, 30–32]. Central neuropathic itch may often present concomitant with neuropathic pain and may be even more dramatic in cases where it occurs in association with cutaneous sensory loss, which combined may lead to incoercible scratching to the point of self-injury [7, 8, 13, 16–18]. 

In the present study, the access to detailed brain imaging provided a unique opportunity for the investigation of the relationship between these systems in humans, shedding light on the discussion regarding plausible mechanisms implicated in the manifestation and perpetuation of chronic neuropathic itch and pain.

### Pain and itch crosstalk in physiological and pathological conditions

It has been consistently observed that painful stimuli suppress itch [5, 9, 33]. Pruriceptors are polymodal and may also respond to algogens [3, 30]. Therefore, the activation of small itch-selective neurons, regardless of stimulus modalities, generates itch, whilst the activation of larger population of pain afferents and pain-mediating neurons in the spinal cord stimulates, via glutamate release signaling, inhibitory neurons to suppress itch [3, 30, 32]. Interestingly, specialized itch projection neurons (detailed below), do not exhibit spontaneous activity as pain projection neurons do, due to active inhibition exerted by pain-processing neurons. Suppressing this inhibition might provoke itch of central origin, without any activation of primary pruriceptive neurons from the skin [3, 30–32, 34, 35]. This suggests that, based on the described physiological interactions, inhibition of pain may produce itch [30].

The pain/itch network crosstalk is also well illustrated through the mechanisms of action of opioids [35]. The administration of morphine inhibits pain at the expense of eliciting itch, which is mediated by the direct activation of distinct isoforms of µ-opioid receptors (MOR) in the spine, but likely also by other indirect mechanisms [30, 35]. Given that, in the present case, thalamic DBS led the patient to experience a dramatic improvement of her otherwise long-standing refractory neuropathic pain, it is plausible that effective suppression of pain via neuromodulation could have also modulated the pain/itch interacting networks, contributing to perpetuation and exacerbation of itch.

### Peripheral pathways and thalamic relays for pain and itch

The underlying neural mechanisms for itch sensation are still not completely understood [31]. Although historically itch was regarded as a weak form of pain, both transmitted by the same type of non-myelinated C-fibres via the lateral spinothalamic tract (STT) to reach the thalamus and somatosensory cortex, this notion has been challenged during the last decades [1, 3, 5]. Torebjörk and Ochoa showed already in the 1980s that, intraneuronal microstimulation of subpopulations of unmyelinated C-fibres could evoke either pain or itch, independently [5]. Furthermore, the increase of frequency and amplitude of stimulation of itch fibers would not change the quality of sensation to pain [5]. Other authors showed that increasing dosages of histamine injection into the skin did not change itch to pain, but led to increased itch sensation [5, 6]. Therefore, the ability to distinguish pain and itch originating from the same region, amongst other evidence, suggests completely different sensory qualities underpinned by distinct interacting neural networks [1, 5].

Although advances in the understanding of the neurobiology of itch have been achieved, questions regarding the mechanisms involved in chronic neuropathic itch remain to be further elucidated [30, 31]. Thus far, pre-clinical and clinical studies support the idea of a network imbalance leading to peripheral and central sensitization of nonhistaminergic itch-signaling neurons, possibly combined with disruption of descending itch modulation pathways [1, 3, 9]. Pruriceptive STT neurons also respond to noxious mechanical and a combination of noxious thermal and chemical stimulation, indicating a polymodal chemonociceptive function [4]. Itch has been shown to be encoded by about one-third of STT neurons; the other two-thirds are nociceptive only [4]. Microantidromic mapping of histaminergic and nonhistaminergic pruriceptive neurons in the primate has shown that these neurons integrate with the STT, terminating in several thalamic nuclei of the lateral, ventral basal and posterior complex [4]. Andrew and Craig et al. [33] demonstrated the existence of specific lamina I itch neurons projecting to the VPI and to the lateral border of VPL, showing that itch is subserved by specific neural networks, distinct from that responsible for pain and temperature information. Nociceptive STT neurons project more often to the posterior portion of the ventral medial nucleus [36], whilst thermoreceptive neurons of the STT have a distinct projection pattern to the posterior thalamus [36]. Davidson et al. [4] corroborated previous findings, showing that both histamine-responsive and nonhistaminergic pruriceptive neurons terminate in the ventral posterior inferior and ventral posterior lateral nuclei, however the latter has also connections with the suprageniculate and geniculate nuclei. Although, it is likely that multiple areas within the thalamus remain relevant, most pruriceptive STT neurons appear to track to the lateral portion of the ventral posterior lateral nucleus, suggesting the prominent role of this area in the neurobiology of pruritus [4]. Detailed imaging available in our case, indeed demonstrated the location of the active stimulating contact in this exact area. This information together with the clinical evidence of stimulation driven itch which objectively improved when the stimulation was ceased unilaterally in the corresponding hemibody but not in the opposite hemibody which continued to receive stimulation, for the first time, provides direct evidence of a “central itch” centre in the lateral portion of the ventral posterior lateral/ventral posteroinferior nuclei.

Thus, in our patient, the mechanism implicated in the development of severe itchy rash was pruritus triggered by stimulation of the lateral aspect of the ventral posterolateral and ventral posterior inferior nuclei, which led to scratching and chronic inflammation of the skin. In addition, subsequently, intracutaneous inflammation caused by incoercible scratching might have led to further neurogenic inflammation and perpetuation of chronic itch, in a vicious cycle [1, 30, 31, 34].

### Thalamocortical circuitry

Several brain regions have been implicated in the pathophysiology and shown to be activated during an itch [1, 3, 9, 10]. These include brain areas involved in (i) recognition and attention to the symptom (thalamus, primary and secondary somatosensory cortices), (ii) associated with emotional awareness and planning of motor response to itch (cingulate and insular cortices), (iii) associated with the subjective sensation of feeling of itch (the medial parietal cortex, posterior cingulate cortex and precuneus) and (iv) related to the execution of motor response, (supplementary, premotor, primary motor cortices, striatum and cerebellum) [1, 3, 4]. Furthermore, the act of scratching during itch has been shown to activate the ventral striatum, medial prefrontal cortex, anterior cingulate end orbitofrontal cortex, which are well-known brain structures associated with pleasure [1, 3, 9].

Thus, another potential mechanism for our observation may relate to the thalamo-cortical projections and somatotopic organization of ascending fibers within the posterior limb of the internal capsule and thalamic peduncles. As previously discussed, specialized itch ascending STT lamina I neurons synapse with thalamic third neurons within the VPL and VPI nuclei, which then project to somatosensory cortical regions, cingulate, insula and anterior cingulate. The thalamic peduncles consist of ascending and descending fibre bundles that detach from the corona radiata and internal capsule to connect thalamic nuclei not only to the cerebral cortex, but to the brainstem, spinal cord and cerebellum [37]. The anterior thalamic peduncle travels along the anterior limb of the internal capsule to connect, projecting to the prefrontal cortex, orbitofrontal and anterior cingulate. The fibres within the superior and posterior thalamic peduncles, connect reciprocally the thalamus to central parietal and occipitotemporal areas of the cortex, whilst the inferior thalamic peduncle runs medially to the posterior limb of the internal capsule, reaching the posterior insula, temporal cortex and the brainstem [4, 33, 37]. Because active contacts were sitting in the lateral border of the thalamus, in close relationship with the internal capsule and thalamic peduncles, the electric current may have not only stimulated the thalamus directly but also orthodromically stimulated these fibers, giving rise to sensory disturbance, interpreted as itch.

Despite of extensive neurological and dermatological investigation, close clinical follow-up and systematic documentation, this observational study has limitations. Firstly, due to the severity of her skin lesions, the patient was kept on previous pharmacological treatment throughout the evaluation period, which might have partially contributed to improvement of itch. Secondly, the evaluation of itch symptoms and skin lesions might have been biased by the open-label design of the study, where both patient and medical staff were aware of which electrode had been temporarily switched off. Finally, regarding limitations intrinsic to the method of image processing and analysis of the position of the active contacts within the thalamus, the neural structures identified by the application of standard and normative stereotactic brain atlas might not correspond exactly to the patient’s specific neuroanatomy.

Pathophysiology of central itch remains complex. A better understanding of the underlying mechanisms involved in the genesis and interactions between pain and itch, may contribute to the further development of future analgesic and antipruritic therapies. Targeted neuromodulation of the peripheral and/or central nervous system has already proven to be highly effective in selected conditions associated with refractory neuropathic pain [38]. Previous works using transcranial direct current stimulation and cutaneous field stimulation have also reported on the potential benefits of neuromodulation in the management of neuropathic chronic itch [39, 40]. The present work sheds light on mechanisms implicated in the manifestation of central itch secondary to direct stimulation of deep brain structures, opening an avenue for further investigation of neuromodulative strategies in the treatment of such challenging conditions.

## Conclusions

Thalamic neuromodulation as cause of neuropathic central itch is a rare condition. We postulate that the lateral portion of the ventral posterolateral and ventral posteroinferior nuclei of the thalamus, play an important role in the neurophysiology of itch in humans. Our findings shed light on the neurobiology of central itch, providing an opportunity to further investigate the sensory thalamus as a potential locus for neuromodulation in the management of chronic neuropathic itch.

## Data Availability

The data that support the findings of this study are included in the article. The manuscript was written following the Strengthening the Reporting of Observational Studies in Epidemiology (STROBE) checklist.

## Abbreviations

DBS: Deep brain stimulation
STT: Spinothalamic tract
VPL: Ventral posterolateral nucleus
VPI: Ventral posteroinferior nucleus
VPM: Ventral posteromedial nucleus
NI: Neuropathic itch
VAS: Visual Analogue Scale
